# Controlling Attention to Nociceptive Stimuli with Working Memory

**DOI:** 10.1371/journal.pone.0020926

**Published:** 2011-06-07

**Authors:** Valéry Legrain, Geert Crombez, André Mouraux

**Affiliations:** 1 Department of Experimental Clinical and Health Psychology, Ghent University, Ghent, Belgium; 2 Institute of Neuroscience, Université catholique de Louvain, Louvain-la-Neuve and Brussels, Belgium; Alexander Flemming Biomedical Sciences Research Center, Greece

## Abstract

**Background:**

Because pain often signals the occurrence of potential tissue damage, a nociceptive stimulus has the capacity to involuntarily capture attention and take priority over other sensory inputs. Whether distraction by nociception actually occurs may depend upon the cognitive characteristics of the ongoing activities. The present study tested the role of working memory in controlling the attentional capture by nociception.

**Methodology and Principal Findings:**

Participants performed visual discrimination and matching tasks in which visual targets were shortly preceded by a tactile distracter. The two tasks were chosen because of the different effects the involvement of working memory produces on performance, in order to dissociate the specific role of working memory in the control of attention from the effect of general resource demands. Occasionally (i.e. 17% of the trials), tactile distracters were replaced by a novel nociceptive stimulus in order to distract participants from the visual tasks. Indeed, in the control conditions (no working memory), reaction times to visual targets were increased when the target was preceded by a novel nociceptive distracter as compared to the target preceded by a frequent tactile distracter, suggesting attentional capture by the novel nociceptive stimulus. However, when the task required an active rehearsal of the visual target in working memory, the novel nociceptive stimulus no longer induced a lengthening of reaction times to visual targets, indicating a reduction of the distraction produced by the novel nociceptive stimulus. This effect was independent of the overall task demands.

**Conclusion and Significance:**

Loading working memory with pain-unrelated information may reduce the ability of nociceptive input to involuntarily capture attention, and shields cognitive processing from nociceptive distraction. An efficient control of attention over pain is best guaranteed by the ability to maintain active goal priorities during achievement of cognitive activities and to keep pain-related information out of task settings.

## Introduction

Pain is more than the subjective experience of unpleasantness associated with a somatic sensation. It is an important biological signal of physical threat that urges escape. As such, nociceptive stimuli have the capacity to involuntarily capture attention and to interfere with ongoing cognitive and behavioral activities in order to allocate resources to handling potential physical threats [1], [2]. Experiments have documented the disruptive effect of pain by revealing that the delivery of a nociceptive stimulus deteriorates the performance of a pain-unrelated task (e.g. [3], [4]). Further studies have shown that the “attentional” context in which the nociceptive stimulus is delivered (i.e., its salience and its relevance), rather than pain per se, determines how ongoing activities are disrupted (see [2], [5]).

Building on this notion, an over-responsive disruptive function of pain has been incriminated in the persistence of chronic pain states in patients who tend to become increasingly attentive to pain-related information [6]. This over-responsiveness can have a negative impact on the cognitive abilities required for daily-life activities [7]. Therefore, it is of primary importance to understand how and to what extent the attention given to nociceptive inputs can be controlled. It was recently hypothesized that the direction of attention away from vs. towards pain-related information is under the influence of working memory [2]. Indeed, the capture of attention by a stimulus is contingent on the similarities shared between the features of the stimulus and the features the individual is attending to perform the task [8]. Because working memory transiently stores and rehearses the information that is relevant for the achievement of current goals, working memory helps to guide the selection of attended targets [9]–[12] and can control involuntary shifts of attention towards irrelevant distracters [13]–[15].

Similar results were found for nociception in a recent study which has shown that nociceptive distracters interfere less with the processing of task-relevant and pain-unrelated visual targets when working memory is rehearsing these targets [16]. In that study, a selective attention paradigm was used in which visual targets were shortly preceded by task-irrelevant somatosensory distracters (see [3]). The somatosensory distracters were innocuous tactile stimuli occasionally and unexpectedly replaced by a novel nociceptive stimulus. The occurrence of the nociceptive stimulus was made novel in order to increase its ability to capture attention and to interfere with the visual task. Indeed, novelty is known to be one of the most determinant factors to capture attention [5], [17]. Therefore, as expected, reaction times to visual targets were slower when the targets were preceded by a novel nociceptive distracter, as compared to targets preceded by a standard tactile distracter [3], [5], [17]. Most interestingly, when working memory was involved in the visual task, the distractive effect produced by the novel nociceptive distracters was suppressed [16]. In that study, the involvement of working memory was obtained by asking participants to not respond according to the features of the current visual target, but according to the features of the visual target presented one trial before [18], [19]. In other words, they were asked to delay their response to each visual stimulus until the next trial and to mentally rehearse the target during the time interval during which the somatosensory distracter occurred. It was thus concluded that actively holding in working memory the features of pain-unrelated relevant stimuli may prevent attention from being captured by nociceptive stimuli [16].

The aim of the present study was to extend previous results [16] and, most importantly, to rule out the possibility that the suppression of distraction observed in the working memory task was due to an increase of general task demands exerted on attentional resource allocation and task performance. Indeed, it is acknowledged that changing task demands can modify the load of attention that is allocated to nociceptive distracters independently of the processes specifically involved in the task, and most previous studies on this topic did not take into account the confounding effect of attentional load (see [20]). Here, to dissociate the specific contribution of working memory to the control of attention from the effects due to general task demands, we used two different working memory tasks, with different effects on task performance relatively to their control conditions. The first one was the same as in our previous study [16] (*1-back discrimination task*), a task where the involvement of working memory is well known to facilitate response latencies [18], [19]. The second task was a task in which participants were asked to match the features of the current visual target to the features of the target presented one trial before (*1-back matching task*) [21]. Unlike the former task, response latencies in this matching task are increased (see [22]). Hence, it was expected that, if working memory is specifically involved in the shielding of task-relevant information, the distraction produced by novel nociceptive stimuli would be reduced in the condition in which the visual task required to rehearse visual target features in working memory as compared to the condition which did not require rehearsing, and that this effect of working memory would be independent of whether general performance was facilitated or deteriorated by the demands of the working memory task.

## Methods

### Participants

Participants were 14 healthy volunteers (mean age 25±4 years; 9 women; 1 left-handed), with normal or corrected-to-normal vision, no prior history of neurological, psychiatric or chronic pain disorders and no current psychotropic or analgesic drug use. Experimental procedures were approved by the Ethics Committee of the Université catholique de Louvain (B40320096449). Written informed consent was obtained from participants.

### Stimuli

Nociceptive somatosensory stimuli were 50-ms pulses of radiant heat generated by a CO_2_ laser (10.6-µm wavelength; Université catholique de Louvain), delivered to the dorsum of left hand, within the sensory territory of the superficial radial nerve. Beam surface on the skin was ∼80 mm^2^. Stimulus energy (M = 700±100 mJ, ranging from 470 to 880 mJ) was adjusted individually to elicit a clear pinprick sensation, perceived as slightly painful, related to the activation of Aδ-fiber skin nociceptors (see [23]). To prevent nociceptor fatigue, sensitization, and skin overheating, the target of the laser beam was slightly displaced after each pulse.

Tactile somatosensory stimuli were 0.5-ms constant current square-wave electrical pulses (DS7 Stimulator, Digitimer Ltd) delivered with a pair of electrodes (0.7-cm diameter, 2.5-cm inter-electrode distance) placed on the left forearm, close to the wrist, over the superficial branch of the radial nerve. Intensity was set at 1.5 times the absolute detection threshold. This intensity (M = 0.89±0.21 mA, ranging from 0.50 to 1.30 mA) was above the threshold of tactile Aβ-fibers, but well below the threshold of nociceptive Aδ- and C-fibers [24].

Because experiments were conducted during two different sessions, we ensured that stimulus intensities did not change between the two sessions, neither for laser stimuli (F_1,13_ = .207, p = .657, η^2^ = .016) and electrocutaneous stimuli (F_1,13_ = .642, p = .437, η^2^ = .047).

Visual stimuli were presented on a 17” CRT monitor placed 70 cm in front of the participant. Stimuli were made of two 6-cm blue (RGB 0*0*255) or yellow (RGB 255*255*0) colored disks displayed on a black background, 3-cm left and right from a white 1.7-cm central fixation cross.

### Procedure

**Figure 1:**
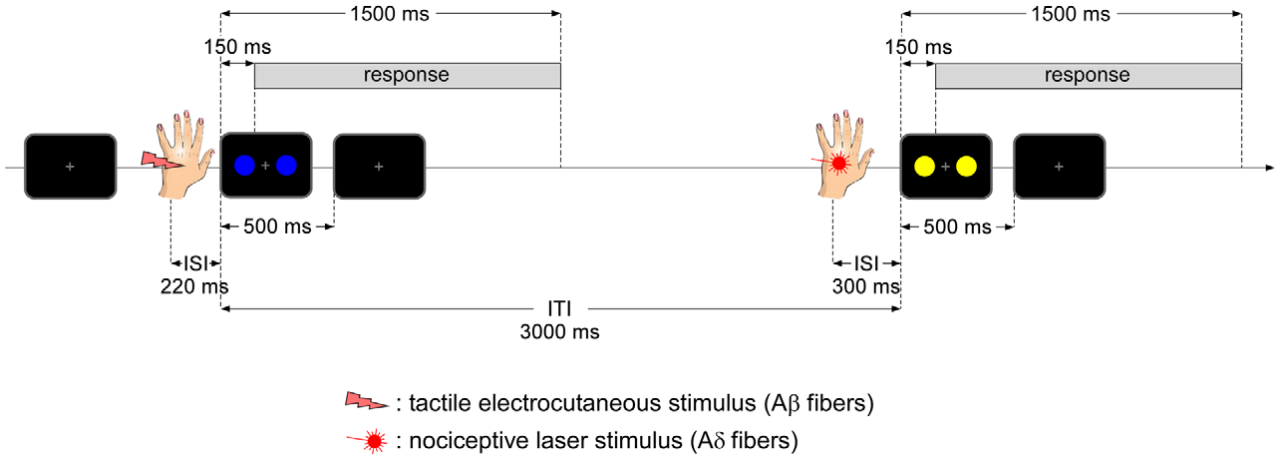
Experimental trials. The experiment started with a grey fixation cross that was present at the center of the screen (black background) during the entire stimulation block. Each trial started with a somatosensory stimulus. Somatosensory stimulus was either a 0.5-ms tactile electrocutaneous pulse applied over the left *nervus radialis* or a 50-ms laser nociceptive pulse applied to the left hand dorsum. Each somatosensory stimulus was followed by a visual stimulus presented briefly during 500 ms and consisting of two 6-cm circles at 4.9° left and right from the fixation cross. The color of the circles was blue (RGB 0*0*255) and/or yellow (RGB 255*255*0). The inter-stimulus time interval (ISI) between the onset of the somatosensory stimulus and the onset of the visual stimulus was 220 ms when the somatosensory stimulus was tactile, and 300 ms when it was nociceptive. The inter-trial time interval (ITI) was 3000 ms measured between the onsets of visual stimuli. Participants were asked to respond to the color of the visual stimuli. Performance was measured within the time window running from 150 to 1500 ms after visual stimulus onset.

**Figure 2:**
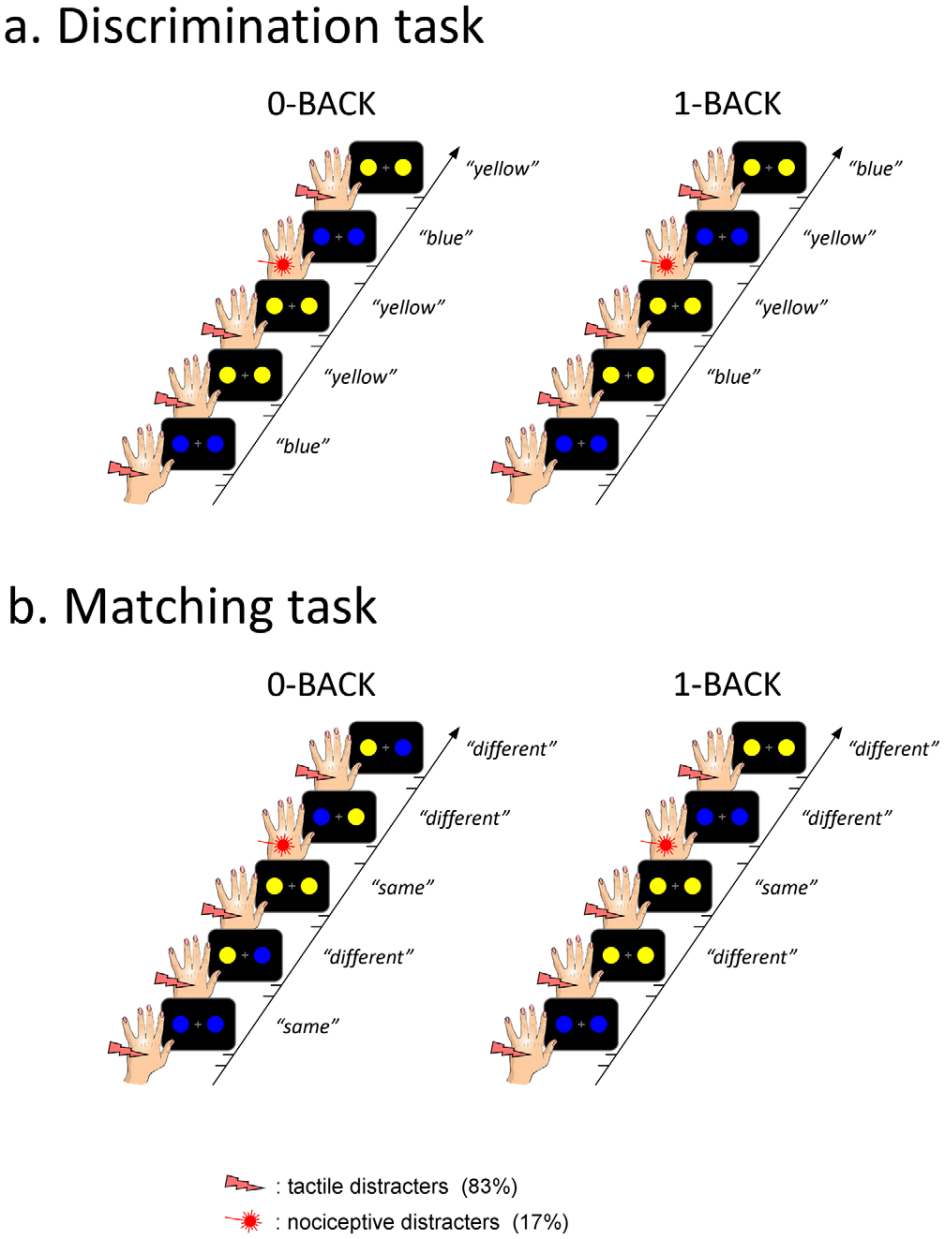
Experimental paradigm. (a) During one of the two sessions, participants were involved in a color discrimination task in which they had to respond according to the color of each visual stimulus constituted of two circles that were either both yellow or both blue. In the 0-back condition, they responded according to the color of the current stimulus. In the 1-back condition, they responded according to the color to the stimulus that was presented one trial before. (b) During the other session, participants performed a color matching task in which they had to respond according to whether the colors of two targets were matched or unmatched. In the 0-back condition, they compared the color of the two circles of the current stimulus, which were matched (yellow-yellow, blue-blue) or unmatched (yellow-blue, blue-yellow). In the 1-back condition, they compared the color of the current stimulus (yellow-yellow, blue-blue) to the color of the preceding stimulus (yellow-yellow, blue-blue). Note that only the 0-back matching task contained stimulus in which colors of the two circles could be different. The visual targets were preceded by a tactile stimulus in 83% of trials, or by a nociceptive stimulus in the remaining 17% of trials.

The experimental design is illustrated in Figures 1 and 2. Participants were presented with 12 blocks, distributed over 2 different sessions (6 blocks per session). Each block consisted of 60 trials. A fixation cross remained at the center of the monitor for the entire duration of a block. Each trial started with a somatosensory stimulus (tactile or nociceptive) shortly followed by a visual stimulus presented briefly during 500 ms. The inter-stimulus time interval (ISI) between the onset of the somatosensory stimulus and the onset of the visual stimulus varied according to the type of somatosensory stimulus, in order to account for the faster conduction velocity of Aβ-fibers conveying the tactile input vs. Aδ-fibers conveying the nociceptive input: ISI was 220 ms for the tactile-visual trials and 300 ms for the nociceptive-visual trials [24]. The inter-trial time interval (ITI) between the onsets of two consecutive visual stimuli was 3000 ms (Figure 1). Fixed temporal parameters were used as random time intervals could have modified stimulus salience [25]. In particular, by disrupting the monotony induced by the constant repetition of standard tactile stimuli, the use of random time intervals might have decreased the salience contrast between the standard tactile stimuli and the novel nociceptive distracters.

Within each block, the trials were delivered in a pseudo-random order, using the following restrictions. To maximize the novelty of the nociceptive vs. tactile distracters, (1) the probability of occurrence was 0.83 for tactile-visual trials (50 trials per block) and 0.17 for nociceptive-visual trials (10 trials per block), (2) nociceptive-visual trials were preceded by at least three tactile-visual trials and (3) the first four trials of a block never included a nociceptive-visual trial. To prevent any preference for a given response, and to prevent any association between the type of nociceptive-visual trial and the type of response, (4) the probabilities of each of the two possible responses were equivalent, (5) each type of somatosensory distracter was equally associated with each type of response, (6) each type of response was equally likely to be preceded by the same or a different type of response, and (7) this equivalence was maintained across the two types of somatosensory distracters.

During one of the two sessions, participants performed a *color discrimination task* (Figure 2a). The color of the two disks constituting the visual target was either both blue or both yellow (i.e. blue-blue, yellow-yellow). Immediately following the onset of the visual target, they were asked to respond according to the color of the current visual target (0-back condition, three blocks) or the color of the visual target presented one trial before (1-back condition, three blocks). During the second session, participants performed a *color matching task* (Figure 2b). In the 0-back condition, participants reported whether the two disks of the visual target were of matching color. The two disks could be either matching (blue-blue, yellow-yellow) or non-matching (yellow-blue, blue-yellow). In the 1-back condition, participants matched the color of the current visual target to the color of the preceding visual target. The two disks of each target were always of the same color (blue-blue, yellow-yellow). The order of the two sessions was balanced across participants.

For all conditions, participants were asked to respond as accurately and as fast as possible. Responses were produced by pressing one of two keys on a numerical keypad with their right middle finger or index finger. They were instructed to keep both fingers on the response keys in order to prevent using the target finger as a proprioceptive or visual clue in the 1-back color discrimination task. They practiced the 1-back task prior to each experimental session with a block of ∼20 visual stimuli without any associated somatosensory stimuli. No ratings for somatosensory stimuli were asked during the experiment in order to not interfere with task instruction since bottom-up attention paradigms require to keep distracters irrelevant for the task [26].

### Analyses

Performance of the visual task was measured by the percentage of errors for response accuracy and by the mean reaction times (RTs) for response speed (excluding the first response of each block, incorrect responses, anticipated responses [RT<150 ms], and missed responses [RT>1500 ms]). This cut-off was chosen according to pre-testing experiment having revealed that reaction times below 150 ms and above 1500 ms are outliers. Tactile-visual trials that immediately followed a nociceptive-visual trial were also not included in the analyses. Eight conditions resulted from the combination of the following three independent variables: *visual task* (discrimination vs. matching), *working memory* (0-back vs. 1-back), and *somatosensory distracter* (frequent tactile vs. novel nociceptive). RTs and percentages of error were analyzed using a 3-factor ANOVA for repeated measures (2*2*2 conditions). When appropriate, contrast analyses were used. Size effects were measured with partial Eta-squared for ANOVAs and Cohen's d for t-tests. Significance level was set at *p<0.05* and was adapted for multiple contrast comparisons.

### Supplementary analyses

Additional analyses were conducted in order to dissociate within each task the more and the less demanding trials. Indeed, in addition to working memory capacities, the n-back paradigm offers measures of executive functions such as updating [21] and conflict resolution [27]. For instance, in the 1-back discrimination task, conflict can occur between the correct response and the current stimulus (e.g. the preceding target was yellow, the expected response was “yellow”, but the current stimulus was blue) [16], [18]. Therefore, task demands could have been increased during some trials in order to solve the interference between the memory template and the current stimulus. Consequently, additional analyses were conducted by separating trials with conflict (difference between the expected response and the color of the current stimulus) and trials without conflict (the expected response and the current color are the same). In the 1-back matching task, conflict could also have occurred, but in a different fashion. During the practice session, it was noticed that some participants tended to associate one response key to one color. Such a trend could have had a detrimental effect on performance, as the correct response was not related to the color of the stimulus, but to whether or not that color matched the color of the preceding stimulus. We suspect that when the color of the visual target was repeated but the associated correct response was to be alternated (e.g. Figure 1, trial #3 of the bottom right illustration), or, conversely, when the color of the visual target was alternated but the associated correct response was unchanged (e.g. trial #5 of the same illustration), this could have been a source of interference requiring additional resources. Consequently, additional analyses were conducted by separating trials with conflict (repetition of the stimulus color combined with alternation of the expected response, and alternation of the stimulus color combined with repetition of the expected response) and trials without conflict (stimulus color and correct response are either both repeated or both alternated). In each new data sample, conflict resolution was tested with an ANOVA conducted with *conflict* (conflict vs. no conflict) and *somatosensory distracter* (tactile vs. nociceptive) as factors.

## Results

### Response accuracy

**Figure 3:**
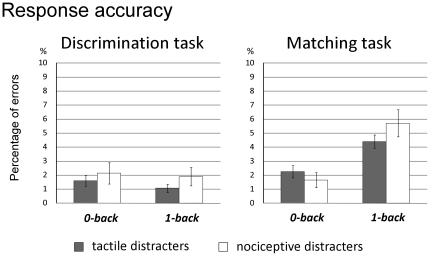
Response accuracy. Percentage of errors to the visual targets according to the task (discrimination vs. matching), the engagement of working memory (0-back vs. 1-back) and the type of somatosensory distracter (novel nociceptive vs. standard tactile). Error bars represent confidence intervals [28].

Participants anticipated 5.33% of the responses in the 1-back condition of the discrimination task, but never anticipated the responses in the other conditions. Overall, participants made very few errors (2.80%). Nevertheless, there was a significant effects of visual task (F_1,13_ = 21.535, p<.001, η^2^ = .624), a significant effect of working memory (F_1,13_ = 8.492, p = .012, η^2^ = .395), as well as a significant interaction between the two factors (F_1,13_ = 17.674, p<.001, η^2^ = .576), suggesting that participants made more errors during the 1-back condition of the matching task as compared to all other conditions (all p<.001, all η^2^≥.627) (Figure 3). There was no significant effect of the type of somatosensory distracter (F_1,13_ = 1.262, p = .282, η^2^ = .088) and no significant interaction with that factor (all p≥.158, all η^2^≤.148).

### Response speed

**Figure 4:**
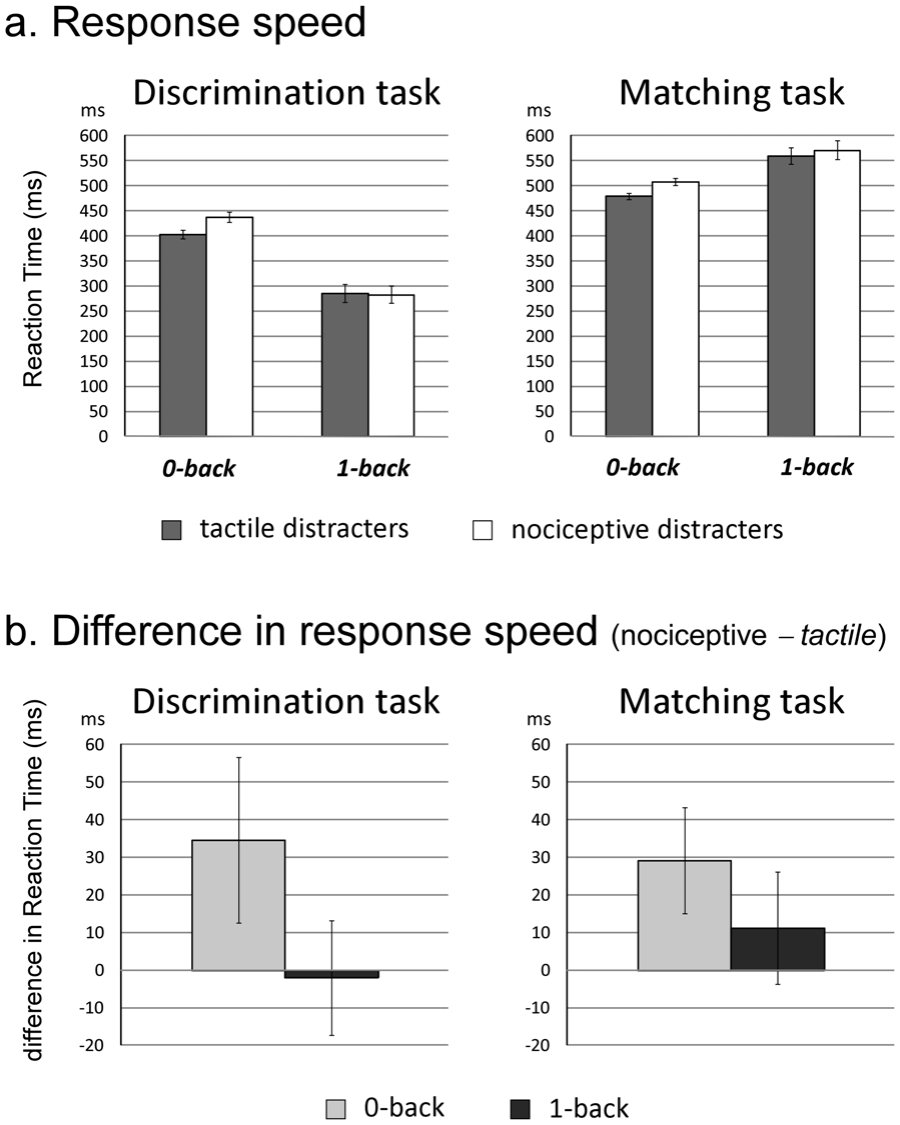
Response speeds. (a) Mean reaction times (RTs) to the visual targets (in milliseconds) according to the task (discrimination vs. matching), the engagement of working memory (0-back vs. 1 back) and the type of somatosensory distracter (novel nociceptive vs. standard tactile). Error bars represent confidence intervals [28]. (b) Distraction indexes assessed by subtracting the mean RTs to the visual targets that followed a standard tactile distracter from the mean RTs to the visual targets that followed a novel nociceptive distracter. Error bars represent standard deviations.

Mean RTs of correct responses are shown in Figure 4a. The ANOVA revealed significant main effects of visual task (F_1,13_ = 83.396, p<.001, η^2^ = .865) and working memory (F_1,13_ = 7.992, p = .014 , η^2^ = .381), as well as a significant interaction between the two factors (F_1,13_ = 52.681, p<.001, η^2^ = .802). This showed that, in the discrimination task, RTs were decreased in the 1-back condition as compared to the 0-back condition (F_1,13_ = 52.602, p<.001, η^2^ = .802), whereas in the matching task, RTs were increased in the 1-back condition as compared to the 0-back condition (F_1,13_ = 16.067, p = .001, η^2^ = .553). In other words, working memory improved performance in the discrimination task, but deteriorated performance in the matching task.

The ANOVA also revealed a significant main effect of the type of somatosensory distracter (F_1,13_ = 14.805, p = .002, η^2^ = .532), and, most importantly, a significant interaction between the type of somatosensory distracter and working memory (F_1,13_ = 12.752, p = .003, η^2^ = .495). In line with our hypothesis, contrast analyses showed that RTs to nociceptive-visual trials were significantly greater than RTs to tactile-visual trials in the 0-back condition but not in the 1-back condition, both during the discrimination task (0-back: t_13_ = −3.231, p = .007, d = .863; 1-back: t_13_ = .482, p = .638, d = .128) and during the matching task (0-back: t_13_ = −5.571, p<.001, d = 1.488; 1-back: t_13_ = −1.804, p = .094, d = .482) (Figure 4b). These effects were not dependent of the task (visual task*somatosensory distracter: F_1,13_ = 0.620, p = .445, η^2^ = .045; triple interaction: F_1,13_ = 3.458, p = .086, η^2^ = .210). Because RT data were not normally distributed in two out of the eight conditions, additional comparisons were performed after transformation of RTs using the reciprocal of latency (i.e. 1/RT). Similar results were obtained: visual task: F_1,13_ = 148.776, p<.001, η^2^ = .920; working memory: F_1,13_ = 31.770, p<.001, η^2^ = .710; somatosensory distracter: F_1,13_ = 11.261, p = .005, η^2^ = .464; task*working memory: F_1,13_ = 68.840, p<.001, η^2^ = .841; working memory*somatosensory F_1,13_ = 20.684, p = .001, η^2^ = .614).

### Supplementary data

Additional analyses on conflict resolution revealed, in the 1-back discrimination task, longer RTs when there was a conflict between the correct response and the color of the current stimulus (F_1,13_ = 5.915, p = .030, η^2^ = .313). There was no significant effect of the type of somatosensory distracter (F_1,13_ = 1.565, p = .233, η^2^ = .107), and no interaction between the two factors (F_1,13_ = .016, p = .902, η^2^ = .001). Similarly, in the 1-back matching task, the conflict between the response and the color of the current stimulus significantly increased RTs (F_1,13_ = 28.563, p<.001, η^2^ = .687). Again, there was no significant effect of the type of somatosensory distracter (F_1,13_ = 1.049, p = .324, η^2^ = .075), and no interaction between the two factors (F_1,13_ = .554, p = .470, η^2^ = .041). Impact of stimulus/response conflict on RTs was confirmed after normalization in both the 1-back discrimination task (F_1,13_ = 6.604, p = .023, η^2^ = .337) and the 1-back matching task (F_1,13_ = 62.249, p<.01, η^2^ = .827) with no influence of the type of somatosensory distracter (all other comparisons: all p≥.101, all η^2^≤.193).

## Discussion

This study reveals that working memory can prevent the distraction triggered by unexpected task-irrelevant novel nociceptive stimuli and, thereby, protect the processing of task-relevant pain-unrelated targets. Indeed, results showed that when the participants were rehearsing the features of the preceding visual targets, the occurrence of a novel nociceptive distracter was less able to disrupt ongoing behavior, and task performance was thereby preserved from a bottom-up shift of attention. The two working memory tasks were taken from previous studies [18], [19], [21], [22], [27]. The involvement of working memory was manipulated by the instruction to delay the response until the presentation of the next trial in the 1-back discrimination task, and to compare features of the current visual stimulus to those of the preceding one in the 1-back matching task. The 1-back discrimination task involves storing and rehearsing the representation of the correct target and/or of the correct response before motor execution. This task reduced response times to visual targets because it allows for some response preparation. However, as motor execution is only allowed at the next trials, the selected target or the selected action has to be maintained and rehearsed in working memory during the time interval between two successive trials in order to avoid decay [16], [18], [19]. Similarly, the 1-back matching task involves storing and rehearsing the visual stimulus. However, unlike the 1-back discrimination task, the selection of the correct response requires processing of the next visual stimulus in order to perform the comparison between the colors of the current and preceding stimuli. Therefore, a memory trace of the preceding stimulus is needed to match its representation to the new stimulus. In addition, in both 1-back tasks, the executive control of working memory (see [29]) is needed to update the content of the store systems after each response in order to prepare the next trial, and is also needed to control proactive interference from other trials [18], [19], [27] (see supplementary data). In both 1-back tasks, working memory was thus active by rehearsing the representation of the relevant visual information during the entire time interval separating two consecutive visual stimuli, that is, during the presentation of the somatosensory distracters. During the 0-back conditions, participants were asked to respond to the visual stimuli directly during their presentation. Thereby, working memory was reset after each trial, and was not needed to perform efficiently the task.

Bottom-up capture of attention represents a mechanism by which attention is shifted away from its current focus towards a stimulus that is sufficiently salient to modify cognitive priorities, even though it is unrelated to ongoing activities [10], [30]. This is particularly the case for stimuli that signal a potential danger for the individual, such as nociceptive stimuli. The capture of attention by salient stimuli can be triggered by mechanisms detecting local contrasts along various physical dimensions in the sensory scene [31] or detecting new inputs and mismatch relative to past events [17]. Regarding nociception, these mechanisms of saliency-detection have been witnessed by increased neural activity in brain areas activated by a nociceptive stimulus [5], [32], [33], particularly when the nociceptive stimulus is presented for the first time [34], [35] or when it is novel and differs among one or more physical features relative to previous stimuli [3], [26], [36]–[38]. An important aspect that should be reminded is that the novelty of a nociceptive stimulus is an important but unspecific feature to capture attention. Indeed, it is important to orient attention in priority to stimuli that signal a mismatch relative to our expectations [10], [17], [30], especially the stimuli that are approaching the body and could eventually represent physical threats [39]. The *unspecificity* of the effect of novelty on the processing of nociceptive stimuli is largely discussed elsewhere [2], [5]. Here, the probability of occurrence of the distracters was used and manipulated in order to make the nociceptive distracters more salient and, thus, to increase their ability to capture attention. The frequent tactile distracters were included to construct a monotonous somatosensory context and to avoid confounding effects between selective attention, i.e. the capacity to focus attention on a subset of information or action, and alerting attention, i.e. a state of stimulus-induced phasic readiness [40]. Therefore, if both the tactile and the nociceptive stimuli were cuing the upcoming occurrence of the visual target (alerting attention), the change from a tactile to a nociceptive distracter was unattended and task-irrelevant, and thus more susceptible to increase attentional capture (bottom-up selective attention) [16].

The control of nociceptive stimuli by attention is an important issue because a large number of studies have demonstrated that attention determines how a nociceptive stimulus will be perceived (see [41]). Decreasing the ability of a nociceptive stimulus to capture attention will affect its processing and, as a consequence, will modify its ability to enter awareness as a pain percept [2]. It was shown recently that nociceptive stimuli can compete for attentional resources with stimuli belonging to other sensory modalities, and that such a competition is accompanied with a proportional change in the magnitude of the brain responses activated by nociceptive stimuli [37], [42]–[44]. Based on current research about attention [8]–[11], [17], [30], [31], [45], a recent review has proposed that the attention paid to a nociceptive stimulus can be controlled by two main factors [2]. The first factor is the attentional set referring to the mental set of stimulus features that are relevant to achieve ongoing cognitive goals [8]. In the present experiment the attentional set was defined by the colors of the visual stimuli in all conditions. Therefore, despite a different mode of response between discrimination and matching tasks, the attentional set was identical across conditions. The second factor is attentional load referring to the effort, in terms of resources allocation, that should be made to achieve the goals adequately [46].

The role of working memory in the control of attention has been mainly supported by studies on visual search [11], [12]. According to competitive models of attention [9], [10], limited access to a full perceptual representation results from competition operations between sensory inputs. At the neurobiological level, competition is expressed by gain control exerted on the responses of neurons representing sensory inputs [9], [45]. In other words, the neural response to a particular stimulus is biased according to its salience (bottom-up filter), as described above, and also according to its relevance (top-down bias). Working memory could be one source of biasing signals, by maintaining active the task-relevant features of the target stimulus for a short period of time [47]. Supporting this view, it was demonstrated that the deployment of selective attention is influenced by the content of working memory [11], [12], [48]–[51]. For instance, studies in the visual domain have shown in dual task paradigms that the direction of attention towards the stimuli delivered in one task, and, therefore, the performance of this task, are influenced by the content of working memory manipulated by the second concomitant task [11], [12], [48], [49], [51]. In other words, when participants are actively rehearsing the features of a stimulus in working memory, attention will be captured by another stimulus if the features of this other stimulus match the features of the stimulus whose representation is currently stored in working memory. Although voluntary control might have an effect on this influence, the guidance of attention by working memory is thought to be rather automatic [12], [50], [51]. A detrimental effect of such automaticity is that if distracters share features with the content of working memory, they are more likely to intrude in the ongoing task and to produce distraction [2], [11], [12]. Conversely, increasing the ability of working memory to keep active the features of the relevant targets prevents intrusion of the distracters and inhibits the shift of attention to them. Indeed, other studies have also shown that manipulating the load of working memory capacity modifies the potential interference from irrelevant distracters [13]–[15].

In the present experiment, the attentional set was defined by the colors of the visual stimuli. Participants were asked to respond to one of the set features in the discrimination tasks (i.e., to press a key corresponding to one of the colors), or to compare two stimuli according to the set features in the matching tasks (i.e., to respond according to whether the colors of two stimuli were matching or not). We showed that maintaining in working memory the target information of the attentional set protected task performance from somatosensory distraction (i.e., suppressed the distractive effect of novel nociceptive stimuli). The innovative point of the present study was to show that suppression of somatosensory distraction could be attributed to the specific involvement of working memory, independently of the attentional overload induced by task demands. Attentional load is generally increased by task difficulty and their demands in terms of attentional resources allocation. As suggested by the overall increase of reaction times and of error rates, the attentional load was probably greater in the 1-back matching task than in the 0-back matching task. During the discrimination task, there was no evidence of greater attentional load for the 1-back condition. Indeed, in the discrimination task, the 1-back condition led to reduced reaction times [16], probably because the task-relevant features of the stimulus could be identified, and the response selected – but also rehearsed – during the time-interval separating the previous and the current target [19]. In contrast, such a response preparation was not possible in the 1-back condition of the matching task which required waiting for the next trial to compare the features of the preceding and the upcoming targets. Participants responded thus more slowly and made more errors in that condition, as typically observed in classic *n*-back matching tasks [22]. Therefore, the observation that, in *both* the discrimination task *and* the matching task, the 1-back condition led to a similar reduction of the disruptive effect of the novel nociceptive distracter indicates that this suppression of distraction was due to the specific involvement of working memory in the control of attention, independently of the effects produced by task demands on attentional load. The absence of effect between conflict and no conflict trials also supports this interpretation. It can be suggested that this reduction of the attentional intrusion of nociceptive distracters induced by engaging working memory is likely to decrease the further processing of the nociceptive stimuli [26] and, as a consequence, is likely to reduce the perception of pain [20].

In addition, the tasks probably differed in terms of the nature of the representation that is stored and rehearsed in working memory: the perceptual representation of the relevant features of the visual stimulus in the 1-back matching task vs. the representation of the correct response in the 1-back discrimination task [16], [19]. This would suggest that working memory is able to control the attention that is allocated to a nociceptive stimulus at different levels of sensory-motor processing.

One important question that remains to be addressed is the ecological relevance of the mechanisms that allow controlling, in a top-down manner, the ability of nociceptive input to capture attention. Indeed, because these inputs signal a potential threat to the body's integrity, it would seem beneficial to immediately attend to these signals regardless of ongoing goal priorities. In fact, an answer to this question may be found in the actual contribution of these mechanisms to the experience of acute and chronic pain. The significance of the top-down control of the disruptive effect of nociceptive input is suggested, for example, by the finding that somatosensory distracters have a more pronounced disruptive effect when participants are frightened by the instruction that the distracters will be delivered at a highly painful level [52] or in subjects having a tendency to catastrophize pain symptoms [53]. Furthermore, it has been proposed that chronic pain symptoms and associated maladaptive behaviors can be reinforced by an excessive attentional profile rendering patients over-attentive to pain- and body-related information [6]. One possible mechanism of this “*hypervigilance to pain*” could be an inability to erase pain-related information from working memory [2]. This interpretation could explain how individual characteristics such as beliefs and worries contribute to amplify the experience of pain [6]. It could also explain the frequent neuropsychological complaints reported by chronic pain patients [7], although it remains unknown whether such deficits result from excessive maintenance of pain-related information in working memory or from a more direct priming effect from persistent nociceptive input.
